# NPS‐2143 inhibit glioma progression by suppressing autophagy through mediating AKT–mTOR pathway

**DOI:** 10.1111/jcmm.18221

**Published:** 2024-03-20

**Authors:** Jia‐Li Nie, Qi Li, Hai‐Tang Yin, Ji‐Hong Yang, Ming Li, Qin Li, Xing‐Hua Fan, Qing‐Qing Zhao, Zhi‐Peng Wen

**Affiliations:** ^1^ Department of Pharmacy Affiliated Hospital of Guizhou Medical University Guiyang P.R. China; ^2^ College of Pharmacy Guizhou Medical University Guiyang P.R. China; ^3^ Centre of Clinical Trials Affiliated Hospital of Guizhou Medical University Guiyang P.R. China; ^4^ Clinical Research Center Affiliated Hospital of Guizhou Medical University Guiyang P.R. China

**Keywords:** autophagy, chemotherapy, glioma, GSEA, NPS‐2143

## Abstract

Gliomas are the most common tumours in the central nervous system. In the present study, we aimed to find a promising anti‐glioma compound and investigate the underlying molecular mechanism. Glioma cells were subjected to the 50 candidate compounds at a final concentration of 10 μM for 72 h, and CCK‐8 was used to evaluate their cytotoxicity. NPS‐2143, an antagonist of calcium‐sensing receptor (CASR), was selected for further study due to its potent cytotoxicity to glioma cells. Our results showed that NPS‐2143 could inhibit the proliferation of glioma cells and induce G1 phase cell cycle arrest. Meanwhile, NPS‐2143 could induce glioma cell apoptosis by increasing the caspase‐3/6/9 activity. NPS‐2143 impaired the immigration and invasion ability of glioma cells by regulating the epithelial–mesenchymal transition process. Mechanically, NPS‐2143 could inhibit autophagy by mediating the AKT–mTOR pathway. Bioinformatic analysis showed that the prognosis of glioma patients with low expression of *CASR* mRNA was better than those with high expression of *CASR* mRNA. Gene set enrichment analysis showed that CASR was associated with cell adhesion molecules and lysosomes in glioma. The nude mice xenograft model showed NPS‐2143 could suppress glioma growth in vivo. In conclusion, NPS‐2143 can suppress the glioma progression by inhibiting autophagy.

## 1 INTRODUCTION

Gliomas are one of the most common tumours in the central nervous system.^1^ There are substantial individual differences in the prognosis of gliomas. The median survival time of low‐grade glioma (LGG) patients is 3–10 years, significantly longer than those of high‐grade gliomas or glioblastoma (GBM).^2^ Temozolomide (TMZ) is the first‐line chemotherapy drug that can substantially prolong the survival time of glioblastoma patients. However, the therapeutic effect of TMZ is influenced by the molecular pathology subtypes of gliomas.^3^ Additionally, previous studies have demonstrated that at least 50% of glioma patients do not respond to TMZ due to innate or acquired resistance.^4^ Therefore, developing novel chemotherapeutic drugs for gliomas is imperative.

Advanced molecular biology and molecular pathology have developed and applied several novel cancer therapeutic strategies in the clinic.^5^, ^6^, ^7^ However, glioma patients have yet to benefit from novel therapeutic drugs, although several drugs have been tested in clinical trials. The blood–brain barrier (BBB) comprises astrocytes, pericytes and perivascular macrophages, which act as the barrier through continuous interaction with these specialized cells.^8^, ^9^, ^10^ In addition to providing necessary conditions for proper neuronal functions and protecting the brain from toxins and pathogens, these specialized cells can prevent the efficient passage of chemotherapy drugs through BBB, which has been considered one of the most important reasons for the failure of glioma drug development.^9^, ^10^, ^11^

Substantial evidence suggests that compound screening may provide new opportunities for cancer therapy.^12^ We established a compound library containing 50 compounds with potential BBB permeability to find small‐molecule compounds as promising glioma therapeutics drugs. Afterward, glioma cells were exposed to these compounds at a final concentration of 10 μM for 72 h, and the compound that showed the most robust anti‐glioma ability was selected for further study. In this study, we found that NPS‐2143, a calcium‐sensing receptor (CASR) antagonist, could inhibit the proliferation of glioma cells and induce cell cycle arrest and apoptosis by suppressing autophagy through mediating the AKT–mTOR pathway.

## 2 MATERIALS AND METHODS

### 2.1 Cell culturing

The human glioma lines U87‐MG, U251 and Hs683 were bought from the Cell Bank of Chinese Academy (Shanghai Institute of Biochemistry and Cell Biology, China Academy of Science). All the glioma cell lines were cultured with Dulbecco's modified Eagle's medium (DMEM) (Gibco, CA, USA) containing 10% fetal bovine serum (FBS) (Biological Industries, USA). In addition, glioma cell lines were cultured in the incubator (Thermo, USA) at 37°C with 5% CO_2_.

### 2.2 Cell proliferation and viability assay

CCK‐8 (Beyotime, China) was used to detect cell proliferation and viability. In brief, glioma cells were seeded in a 96‐well plate at 1000 cells/well density and cultured for 12 h. After treatment with indicated conditions, the culture medium of gliomas was replaced with 100 μL DMEM containing 10 μL CCK‐8 and then incubated at 37°C for 1 h in the dark. The optical density (OD) values were determined at 450 nm using a multi‐well spectrophotometer (Bio‐Rad, USA).

### 2.3 Colony forming assay

Briefly, the glioma cells were seeded in a 6‐well plate at 1000 cells/well density and cultured overnight. The glioma cells were cultured with the indicated concentration of NPS‐2143 for 21 days. After washing with PBS, the cells were fixed with 4% (v/v) paraformaldehyde for 15 min, then stained with 0.1% (w/v) crystal violet for 10 min. After washing with PBS three times, the cell plates were photographed and the number of colonies was counted.

### 2.4 Cell cycle analysis

After treatment with indicated conditions, the glioma cells were digested and fixed with pre‐cool 70% ethyl alcohol overnight in a freezer at −20°C. The cells were centrifuged at 1000 *g* for 5 min at 4°C. After discarding the supernatant, the pellet was re‐suspended with 400 μL of PBS containing 20 μL PI (1 mg/mL), 2 μL RNase A (10 mg/mL) and 0.4 μL Triton‐X and incubated at 37°C for 30 min in the dark. After that, the cell samples were analysed using flow cytometer (Beckman Coulter, USA).

### 2.5 Western blot

After treatment with indicated conditions, the glioma cells were disrupted in RIPA lysis buffer on ice for 30 min. Lysate was obtained by centrifuging at 12,000 *g* for 5 min at 4°C. The sample's protein concentration was quantified using the BCA Protein Assay Kit (Beyotime, China). After that, SDS‐PAGE protein loading buffer was added to lysate with a ratio of 1:4 (v/v) and denatured in water at 99°C for 10 min. 20 μg of protein samples were added to the SDS‐PAGE and separated by electrophoresis. After transferring, the PVDF membrane (Millipore, USA) was blocked with 5% non‐fat milk for 2 h at room temperature. Afterward, PVDF membranes were incubated with the corresponding primary antibodies at 4°C overnight. Next, the PVDF membranes were washed with TBST buffer for 5 min three times at room temperature and incubated with corresponding secondary antibodies at room temperature for 1 h. The blot bands were visualized with ECL reagent (Beyotime, China).

### 2.6 EdU proliferation assay

EdU proliferation assay was performed using EdU 488 Kit (Beyotime, China). Briefly, the glioma cells were cultured with EdU reagent for 2 h at 37°C, fixed with 4% (v/v) paraformaldehyde for 15 min, incubated with 0.3% (v/v) Triton X‐100 for 15 min and then added to Click Additive Solution. After washing with PBS, a fluorescence microscope was used to observe the EdU‐positive cells.

### 2.7 Cell apoptosis analysis

Cell apoptosis assay was performed using Annexin V‐FITC/PI Kit (Beyotime, China). After treatment with indicated condition, the glioma cells were dissociated with trypsin. Then, the cell suspension was centrifuged at 1000 *g* for 5 min. After discarding the supernatant, the pellet was re‐suspended with 200 μL staining buffer with 5 μL Annexin V‐FITC and 10 μL PI and then incubated at 37°C for 30 min in the dark. After that, the cells were assayed by flow cytometer (Beckman, USA).

A Caspase‐3/6/9 activity Kit (Beyotime, China) was used to determine the caspase‐3/6/9 activity of glioma cells. Briefly, the glioma cells were dissociated by trypsin and incubated with lysis buffer on ice for 15 min. The lysate was centrifuged at 15,000 *g* for 15 min at 4°C. Bradford Protein Assay Kit (Beyotime, China) was used to detect the protein concentration of the supernatant. After that, the optical density (OD) values were determined at 405 nm using a multi‐well spectrophotometer (Bio‐Rad, USA). Moreover, the caspase‐3/6/9 activity was normalized by protein concentration.

### 2.8 Immigration and invasion assay

For the wound‐healing assay, the glioma cells treated with NPS‐2143 were scratched with the 10 μL pipette tip to create a wound. The wound‐healing images were captured at the indicated time by microscope. For transwell and invasion assay, the glioma cells treated with NPS‐2143 were re‐suspended with 100 μL serum‐free DMEM and seeded to the upper well of transwell chamber coating or not coating with Matrigel (Corning, USA), and 500 μL DMEM containing 10% FBS was added to the lower chamber. After culturing for 24 h, the upper surface of the membrane was wiped, and the cells on the lower membrane were fixed with paraformaldehyde and then stained with crystal violet. Following washing with PBS three times, the invading cells were photographed by microscope.

### 2.9 Bioinformatic analysis

The influence of *CASR* expression on the overall survival (OS) and recurrence‐free survival (RFS) of glioma patients was analysed using the platform of gene expression profiling interactive analysis (GEPIA).^13^ Glioma expression data were used to predict the functions of *CASR* by gene set enrichment analysis (GSEA).^14^

### 2.10 Nude mice xenograft

The Ethics Committee of the Affiliated Hospital of Guizhou Medical University approved the protocol of this animal study (Approval number: 2020 Ethical Review No. 113, date: 8 April 2020). Male BALB/C nude mice aged 4–5 weeks were purchased from Vital River Laboratory Animal Technology (Beijing, China) and maintained at the specific pathogen‐free laboratory animal room at the Clinical Medical Research Centre of Affiliated Hospital of Guizhou Medical University with a light/light cycle of 12 h and free access to food and water. All the nude mice were cared for according to the institutional guidelines for animal care. U87‐MG cells were dissociated by trypsin and resuspended at a density of 1.0 × 10^8^ cells/mL. After that, U87‐MG cells were subcutaneously injected into the flank of nude mice (100 μL/mice). Body weight and tumour volume were recorded every 2 days, and tumour volume was calculated using the formula volume = length × width^2^/2 (mm^3^). When tumour volumes reached approximately 100 mm^3^, the mice were randomly divided into the control group (corn oil) and the NPS‐2143 group (80 μM/kg). DAS was administrated to nude mice every 2 days three times.

### 2.11 Statistical analysis

Data were presented as the mean ± standard deviation (mean ± SD). The Student's *t*‐test was used for the statistical analysis between the two groups. One‐way ANOVA with Tamhane *post hoc* was used for comparisons of different groups. The chi‐squared test was performed to compare the variable's data among different groups. All the statistical analysis was performed using SPSS software (IBM, USA, version 16.0). *p*‐Value <0.05 was thought to be statistically significant in this study.

## 3 RESULTS

### 3.1 Compound screening

**FIGURE 1:**
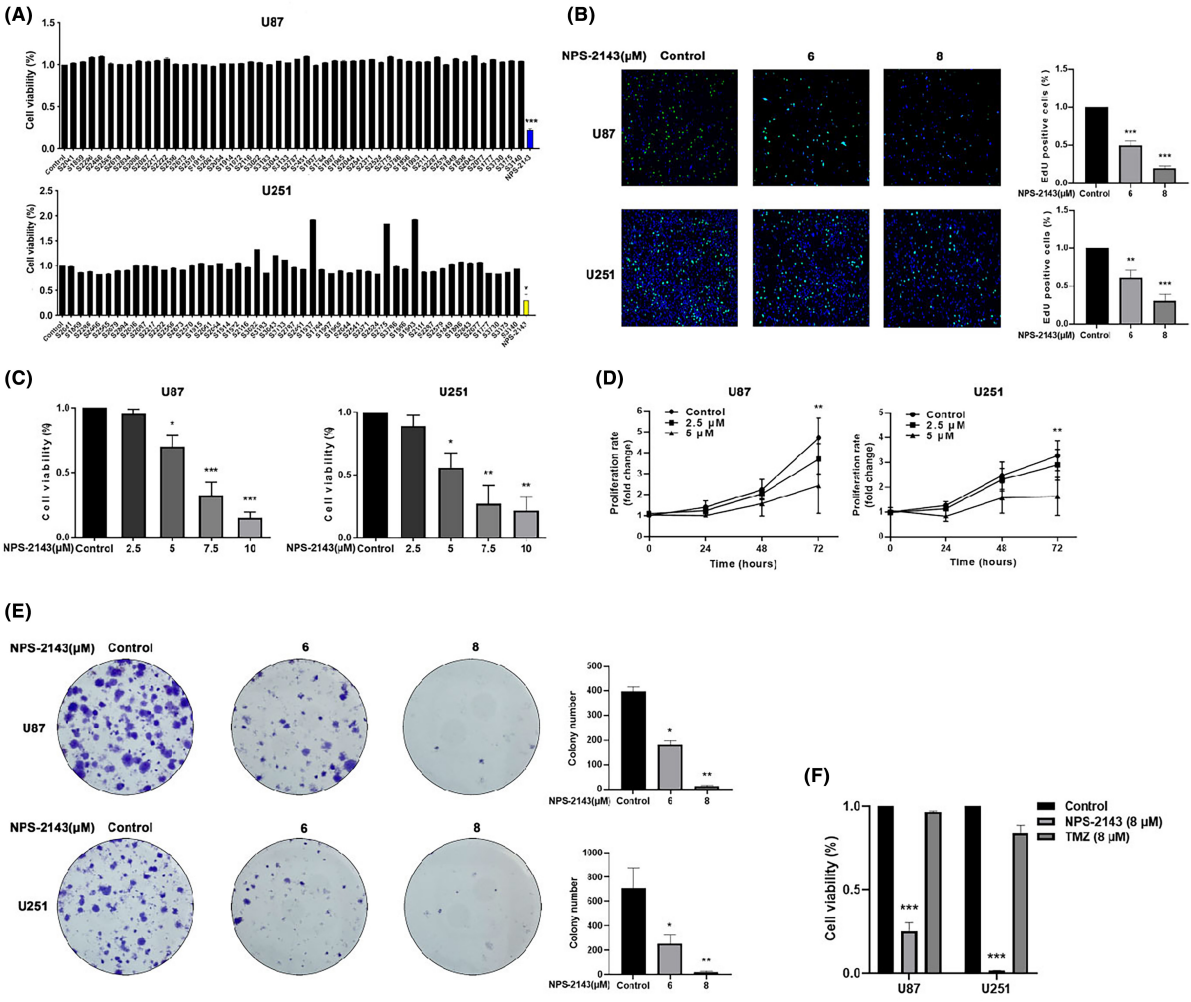
NPS‐2143 suppressed the proliferation of glioma cells. (A) NPS‐2143 showed the strongest anti‐glioma ability among 50 compounds. (B) EdU assay showed that NPS‐2143 significantly decreased the percentage of EdU‐positive U87‐MG and U251 cells. (C, D) CCK‐8 assay indicated that NPS‐2143 obviously decreased the proliferation of glioma cells. (E) NPS‐2143 impaired the clone formation ability of glioma cells. (F) CCK‐8 assay indicated that the cytotoxicity of NPS‐2143 was stronger than TMZ. *p < 0.05, compared with control group; **p < 0.01, compared with control group; ***p < 0.001, compared with control group.

In order to screen novel compounds for the treatment of glioma, a compound library containing 50 compounds was established and purchased from Selleck Chem. All the compounds were dissolved in DMSO, and the U87‐MG and U251 cells were treated with these compounds at the final concentration of 10 μM for 72 h, respectively. NPS‐2143 was selected for further investigation due to its potent anti‐glioma capability (Figure 1A). EdU assay indicated that NPS‐2143 remarkably impaired the proliferation of glioma cells (Figure 1B). Additionally, our results demonstrated that NPS‐2143 could significantly suppress the proliferation of glioma cells (Figure 1C,D). A colony formation assay was performed to evaluate the influence of NPS‐2143 on the long‐term proliferation of glioma cells. As shown in Figure 1E, our results showed that the number of colonies in the NPS‐2143 group was obviously lesser than that in the control group. Moreover, the cytotoxicity of NPS‐2143 and TMZ to glioma cells were compared. As shown in Figure 1F, the anti‐glioma ability of NPS‐2143 was obviously stronger than TMZ.

### 3.2 NPS‐2143 disturbed the cell cycle and related proteins expression in glioma cells

**FIGURE 2:**
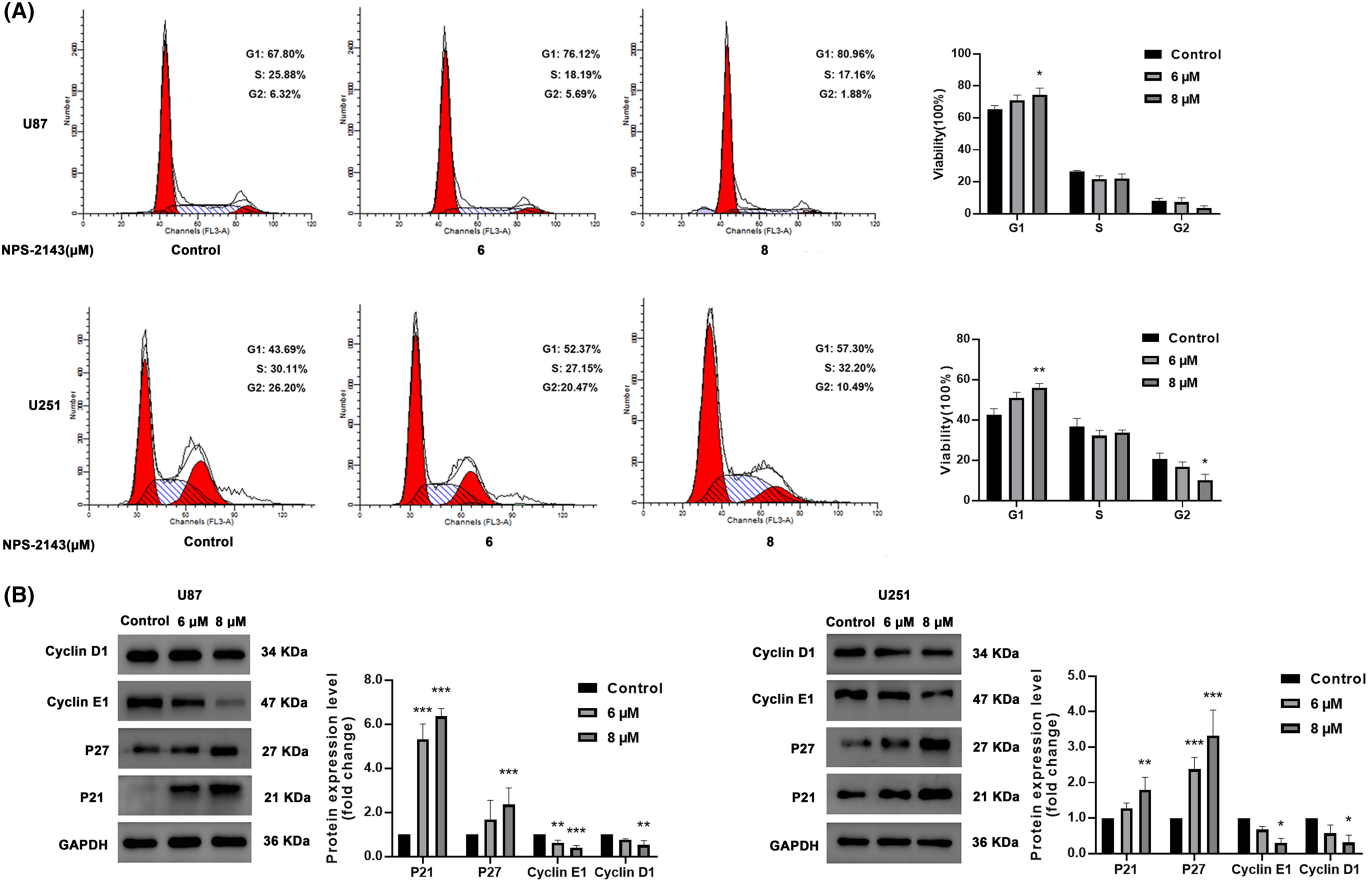
NPS‐2143 induced G1 cell cycle arrest in glioma cells. (A) The proportion of G1 phase glioma cells was significantly elevated in the NPS‐2143 group. (B) Change in protein level of cyclin D1, cyclin E1, P21 and P27. *p < 0.05, compared with control group; **p < 0.01, compared with control group; ***p < 0.001, compared with control group.

Cell cycle arrest might be involved, at least partly, in the NPS‐2143‐induced inhibition of glioma proliferation. Therefore, the cell cycle distribution of glioma cells treated with NPS‐2143 was analysed. Our results showed that in both U87‐MG and U251 glioma cells, the proportion of G1 phase cells in the NPS‐2143 group was significantly higher than those in the control group (Figure 2A). Western blot analysis showed that NPS‐2143 significantly increased the protein expression of P21 and P27 while decreasing the expression of Cyclin D1 and Cyclin E1 (Figure 2B). These results demonstrated that NPS‐2143 resulted in G1 phase cell cycle arrest in glioma cells.

### 3.3 NPS‐2143 induced apoptosis of glioma cells

**FIGURE 3:**
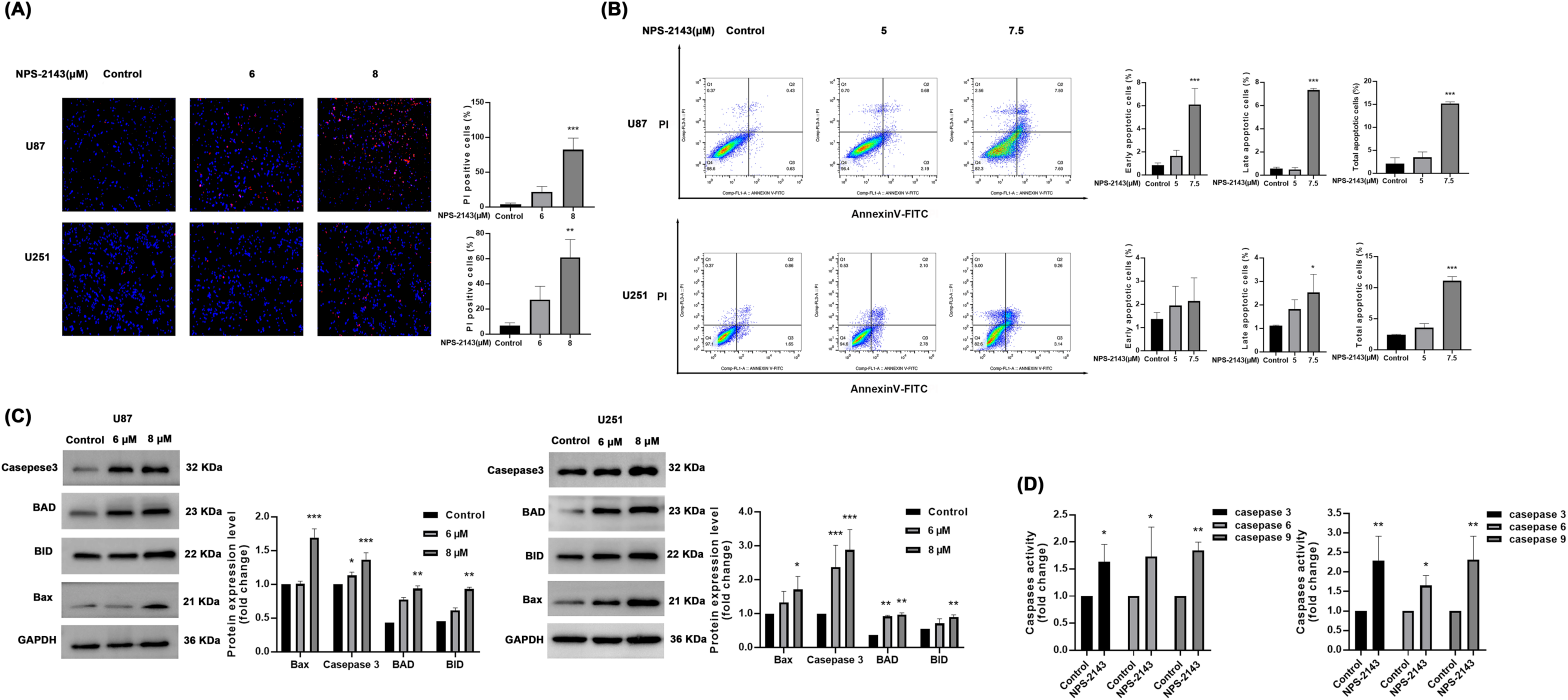
NPS‐2143 induced apoptosis of glioma cells. (A) PI/Hoechst 33343 staining assay demonstrated that NPS‐2143 significantly increased PI‐positive U87‐MG and U251 cells. (B) Cytometry analysis revealed that the apoptotic glioma cells were significantly higher in the NPS‐2143 group than those in the control group. (C) Changes in protein level of caspase 3, BAD, BID and Bax. (D) Caspase activity assay showed that NPS‐2143 markedly increased the activity of Caspase‐3/6/9. *p < 0.05, compared with control group; **p < 0.01, compared with control group; ***p < 0.001, compared with control group.

PI/Hoechst 33342 staining assay demonstrated that the proportion of PI‐positive cells in the NPS‐2143 group was observably higher than that in the control group (Figure 3A), suggesting that NPS‐2143 could cause the death of glioma cells. To further validate whether NPS‐2143 induces apoptosis in U87‐MG and U251 cells, the flow cytometer analysis of annexin V‐FITC/PI was performed. As shown in Figure 3B, the apoptotic cells were all remarkably increased in glioma cells treated with NPS‐2143. Western blot analysis showed that the pro‐apoptosis proteins Bax, BID, BAD and caspase‐3 were significantly higher in the NPS‐2143 group than that in the control group (Figure 3C). In addition, NPS‐2143 could increase the activity of caspases‐3/6/9 in both U87‐MG and U251 cells (Figure 3D). The above results consistently indicated that NPS‐2143 could cause glioma cell death by inducing apoptosis through increasing caspases‐3/6/9 activity.

### 3.4 NPS‐2143 impaired the migration and invasion of glioma cells by regulating epithelial–mesenchymal transition

**FIGURE 4:**
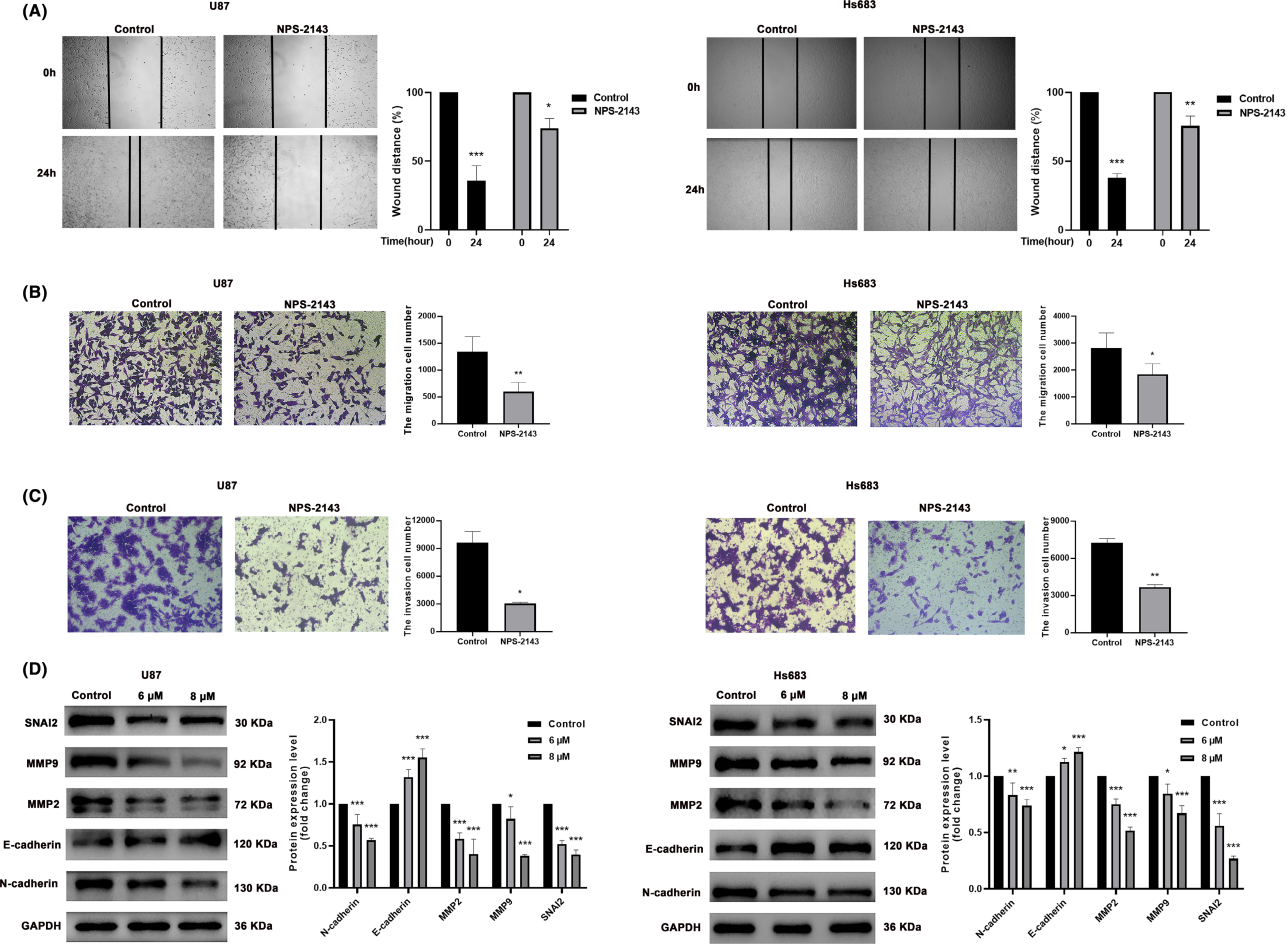
NPS‐2143 inhibited immigration and invasion of glioma cells. (A) Wound heal assay indicated that NPS‐2143 suppressed the immigration ability of glioma cells. (B) Transwell assay showed that NPS‐2143 inhibited the immigration ability of glioma cells. (C) Invasion assay showed that NPS‐2143 suppressed the invasion ability of glioma cells. (D) Changes in the protein level of SNAI2, MMP‐9, MMP‐2, E‐cadherin and N‐cadherin in U87‐MG and Hs683 glioma cells. *p < 0.05, compared with control group; **p < 0.01, compared with control group; ***p < 0.001, compared with control group.

Enhanced migration and invasion abilities of glioma cells were the major factors that caused recurrence in post‐operative patients. Next, we investigated the effect of NPS‐2143 on the migration and invasion of glioma cells. As shown in Figure 4A,B, the wound‐heal and transwell assay results showed that NPS‐2143 significantly decreased the migration ability of glioma cells. Our results also demonstrated that NPS‐2143 could also obviously suppress the invasion ability of glioma cells (Figure 4C). Epithelial–mesenchymal transition (EMT) is a necessary process that controls cell polarity and adhesiveness, which has been implicated in the invasion and infiltration of various cancers. Therefore, the influence of NPS‐2143 on the expression of EMT signal pathway marker proteins was investigated. Western blot analysis demonstrated that NPS‐2143 obviously decreased the protein expression of SNAI2, MMP2, MMP9 and N‐cadherin and increased the expression of E‐cadherin (Figure 4D). These results consistently indicated that NPS‐2143 might interfere with the migration and invasion ability of glioma cells by regulating EMT.

### 3.5 NPS‐2143 inhibited autophagic flux through the AKT–mTOR pathway

**FIGURE 5:**
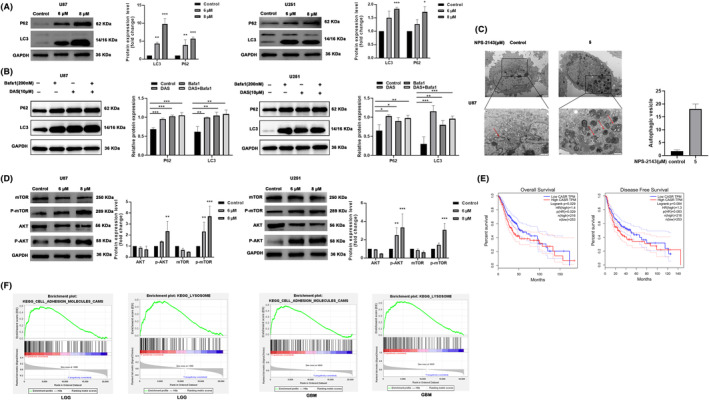
NPS‐2143 inhibited the late stage of autophagy in glioma cells. (A, B) Changes in the protein level of LC3‐II, LC3‐II/LC3‐I and P62. (C) TEM observed the autophagic vacuoles in glioma cells. (D) Changes in protein level of AKT, p‐AKT, mTOR and p‐mTOR. *p < 0.05, compared with control group; **p < 0.01, compared with control group; ***p < 0.001, compared with control group. Bioinformatic analysis of *CASR* in glioma. (E) The effect of *CASR* on the prognosis of glioma patients. (F) GSEA highlights the positive association of *CASR* expression level and cell adhesion molecules and lysosomes.

Our results demonstrated that NPS‐2143 could inhibit the progression of glioma. However, the underlying mechanism was unclear. Previous studies indicated that NPS‐2143 could inhibit autophagy, an important biological process degrading organelles and proteins, which might exert complicated functions in various tumours. Therefore, we further investigated the influence of NPS‐2143 on autophagy activity in glioma cells. As shown in Figure 5A, NPS‐2143 significantly increased the protein expression of LC3‐II, LC3‐II/I and P62, indicating that NPS‐2143 inhibited autophagy in glioma cells. Autophagic flux assay was performed to further investigate the influence of NPS‐2143 on autophagy in glioma cells. As shown in Figure 5B, the LC3‐II expression level was remarkably elevated in DAS, BafA1 and DAS+BafA1 groups, respectively, meanwhile the expression of LC3‐II in DAS+BafA1 group was not higher than that in DAS or BafA1 groups (Figure 5B). Consistently, transmission electron microscope (TEM) results showed that the number of autophagic vacuoles was increased in glioma cells treated by NPS‐2143 (Figure 5C). These results further demonstrated that NPS‐2143 impaired the autophagic flux at the late stage. To further investigate the molecular mechanism underlying the effect of NPS‐2143 on autophagy, the protein levels of AKT, p‐AKT (Ser‐473), mTOR and p‐mTOR (Ser‐2448) were evaluated. Our results showed that NPS‐2143 significantly increased the protein level of p‐AKT and p‐mTOR (Figure 5D). These results indicated that NPS‐2143 might inhibited autophagy by mediating the AKT–mTOR signal pathway.

Previous studies indicated that CASR is the pharmacological target of NPS‐2143. Therefore, we further investigated the role of CASR in glioma progression by using bioinformatics. We found that the overall survival (OS) of glioma patients with a high expression level of *CASR* was significantly longer than that with a low level of *CASR*. However, we found no apparent association observed between the expression of *CASR* and recurrence‐free survival (RFS) in glioma patients (Figure 5E). In addition, our results showed that the level of *CASR* mRNA was significantly positively correlated with the signalling pathways of cell adhesion molecules and lysosomes in low‐grade glioma and glioblastoma by using GSEA, which was similar to our previous results. (Figure 5F).

### 3.6 NPS‐2143 suppress glioma growth in a xenograft mouse model

**FIGURE 6:**
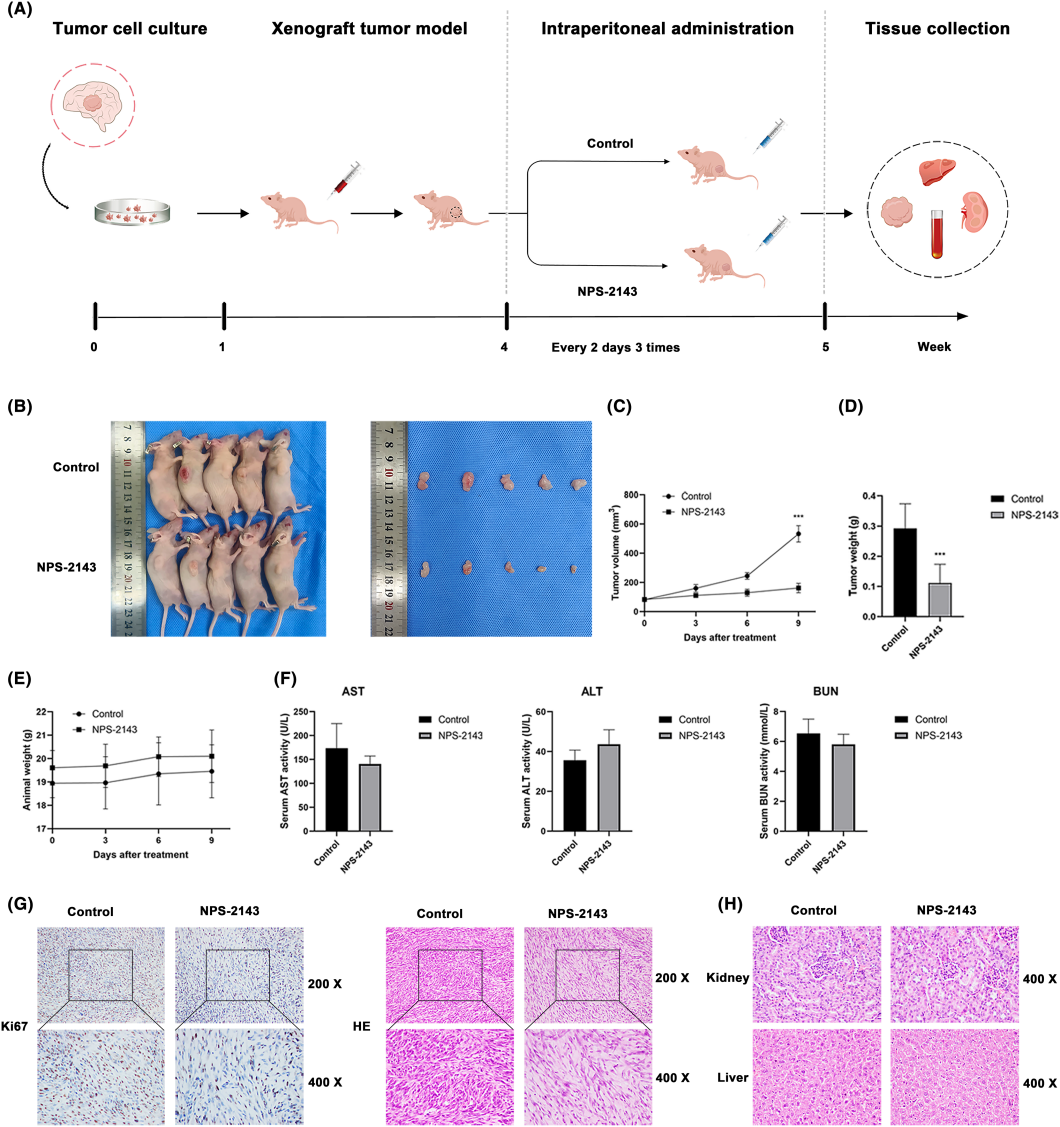
NPS‐2143 suppressed glioma growth in the xenograft nude mice model. (A) The schedule of nude mice xenograft experiment. (B) Photos of nude mice and glioma tissues. (C) Nude mice body weight. (D, E) NPS‐2143 suppressed the glioma tissue volume and weight. (F) Biochemical parameters of liver and kidney function. (G, H) HE and IHC staining analysis of glioma tissues, liver and kidney.

In order to confirm the effects of NPS‐2143 on glioma growth in vivo, a xenograft nude mice model was established. Until the tumour volume reached about 100 mm^3^, the mice were randomly divided into the control group and the NPS‐2143 group, and the mice were intraperitoneally injected with corn oil or NPS‐2143 every 2 days three times, respectively (Figure 6A). Our results showed that NPS‐2143 remarkably suppressed the volume and weight of glioma tissues but did not influence mice body weight (Figure 6B–E). Meanwhile, our results indicated that there was no noticeable difference in ALT, AST and BUN between the control group and the NPS‐2143 group (Figure 6F). IHC and HE staining results suggested that the Ki‐67 protein level was significantly decreased in the NPS‐2143 group, compared with the control group (Figure 6G). In addition, our results also showed that there was no significant difference in the morphology of liver and kidney cells (Figure 6H).

## 4 DISCUSSION

As one of the most common central nervous system malignant tumours in adults, the prognosis of patients with glioblastoma is gloomy, even after receiving surgery, chemotherapy and radiotherapy.^3^ Therefore, it is urgent to develop novel compounds to treat glioma. The present study demonstrated that NPS‐2143 showed the strongest anti‐glioma ability among 50 candidate compounds, and NPS‐2143 could inhibit the growth and invasion, induced cell cycle arrest and apoptosis of glioma by suppressing autophagy through mediating the AKT–mTOR pathway.

NPS‐2143 was a selective antagonist of CASR, which could inhibit CASR and then regulate calcium and bone metabolism.^15^ NPS‐2143 showed therapeutic effects on several types of cancer, but the role of NPS‐2143 in glioma was still unknown.^16^, ^17^, ^18^ In the present study, we first demonstrated that NPS‐2143 could inhibit the proliferation of human glioma cell lines. In order to investigate whether NPS‐2143 suppressed glioma proliferation by interfering with the cell cycle, the proportion of different cell cycle phases was analysed by using a flow cytometer. Our results showed that NPS‐2143 could induce G1 phase cell cycle arrest in glioma cells. Cell cycle transition was fine‐regulated by cyclins, cyclin‐dependent kinases (CDK) and CDK inhibitors.^19^ Our results demonstrated that the protein expression of Cyclin E1 and Cyclin D1 were obviously decreased, while the protein expression of P21 and P27 were significantly increased in glioma cells treated with NPS‐2143. Next, we further investigated the effect of NPS‐2143 on apoptosis in glioma cells. Our results suggested that NPS‐2143 could promote apoptotic glioma cells, which were proved by flow cytometer results, PI/Hoechst 33342 staining and western blot analysis. Additionally, we demonstrated that NPS‐2143 induced glioma cell apoptosis by increasing the caspase‐3/6/9 activity.

Pathologically, most gliomas (Grade II to Grade IV) are characterized by diffuse infiltration growth, which often limits total surgical resection and contributes to the risk of recurrence.^20^ Suppressing the glioma cells' migration and invasion ability may improve the prognosis of patients. Our results showed that NPS‐2143 could inhibit the migration and invasion of U87‐MG and Hs683 cells, which were identified by the wound healing assay, transwell assay and invasion assay. EMT is an essential molecular biological process that results in the epithelial cell loss of cell polarity and adhesiveness and then transforming into mesenchymal cells, which could influence the migration and invasion of cancer cells.^21^ Previous studies demonstrated that EMT was also involved in the migration and invasion of glioma cells.^22^ In the present study, western blot analysis results showed that the protein expression of SNAI1, MMP‐9, MMP‐2 and N‐cadherin was significantly decreased. In contrast, the protein expression of E‐cadherin was significantly increased in glioma cells treated with NPS‐2143. These results suggested that NPS‐2143 could inhibit the immigration and invasion ability of glioma cells by regulating the biological process of EMT.

Autophagy is an evolutionally conserved catabolic biological process that disposes of the damaged organelles and proteins, which is closely involved in cancer and related therapeutic response.^23^, ^24^ The double‐edged sword effects of autophagy showed controversial and complicated influences on tumorigenesis and cancer progression.^25^ Our previous study indicated that inhibited autophagic flux by ablation of autophagy‐related genes could suppress the proliferation and induce the apoptosis of glioma cells.^26^ CASR is a G protein‐coupled receptor family member responsible for calcium homeostasis.^27^ CASR exerted complicated functions in prostate, breast and colorectal cancer by regulating calcium homeostasis and inflammation.^28^, ^29^, ^30^ Previous studies have shown that CASR could regulate autophagy in mammalian cells and that activation or overexpression of CASR induced autophagy, whereas inhibition or knockdown of CASR suppressed autophagy, which might be the molecular mechanism by which CASR regulates cancer.^31^, ^32^, ^33^ Therefore, we speculated that NPS‐2143, the selective CASR antagonist, also might exert anti‐glioma ability by suppressing autophagy. In the present study, we demonstrated that the expression of LC3‐II, LC3‐II/I and P62 was significantly increased in glioma cells treated with NPS‐2143, which suggested that NPS‐2143 might suppress the late stage of autophagy. To confirm this finding further, TEM was performed to observe the autophagic vacuoles in glioma cells after treatment with NPS‐2143. Our results showed that the number of autophagic vacuoles was increased in glioma cells treated with NPS‐2143, which further suggested that NPS‐2143 might impair the late stage of autophagy. Previous study suggested that the AKT–mTOR signal pathway was vital in regulating autophagy in cancer cells.^34^ In order to further investigate the signal pathway underlying the influence of NPS‐2143 on autophagy, the protein levels of p‐AKT and p‐mTOR were analysed. In our study, the NPS‐2143 increased the p‐AKT/AKT and p‐mTOR/mTOR ratio. These results demonstrated that NPS‐2143 might suppress the malignant behaviour of glioma cells by inhibiting autophagy by activating the AKT–mTOR signal pathway. Additionally, Maria Høyer‐Hansen et al. demonstrated that intracellular free cytosolic calcium was a potent autophagy inducer,^35^ which reminded that NPS‐2143 might suppress autophagy in glioma by regulating cytosolic calcium through inhibiting CASR. However, further study should be performed to investigate this hypothesis.

Using the online GEPIA platform, we further explore the potential bio‐functions of CASR in glioma. Our results indicated that the overall survival of glioma patients was negatively correlated with the expression of *CASR* levels. In addition, GSEA revealed that cell adhesion molecules and lysosomes were remarkably enriched in the low‐grade glioma and glioblastoma samples with higher *CASR* levels, respectively. Cell adhesion molecules are a subset of cell surface proteins comprising several families, such as the immunoglobulin superfamily of cell adhesion molecules, cadherins, integrins and the superfamily of C‐type lectin‐like domains protein. Substantial studies have shown that cell adhesion molecules were pivotal for stemness, aggressiveness and invasion of glioma.^36^, ^37^ Lysosomes are the core in autophagy process, the degradation of substrates is dependent on lysosomes in the all types of autophagy. Impeded lysosome‐mediated substrate degradation resulted in the inhibition of autophagy at the late stage. The bioinformatics analysis results correspond with our findings. Additionally, Chun Wang and Shu‐Jin Du's studies demonstrated that NPS‐2143 had a neural protection effect in rat models, which suggests that NPS‐2143 might have the permeability of BBB.^38^, ^39^ However, we did not investigate the BBB permeability of NPS‐2143 in intracranial glioma mice mode. The anti‐glioma ability, BBB permeability and safety of NPS‐2143 should be further validated in future studies.

**FIGURE 7:**
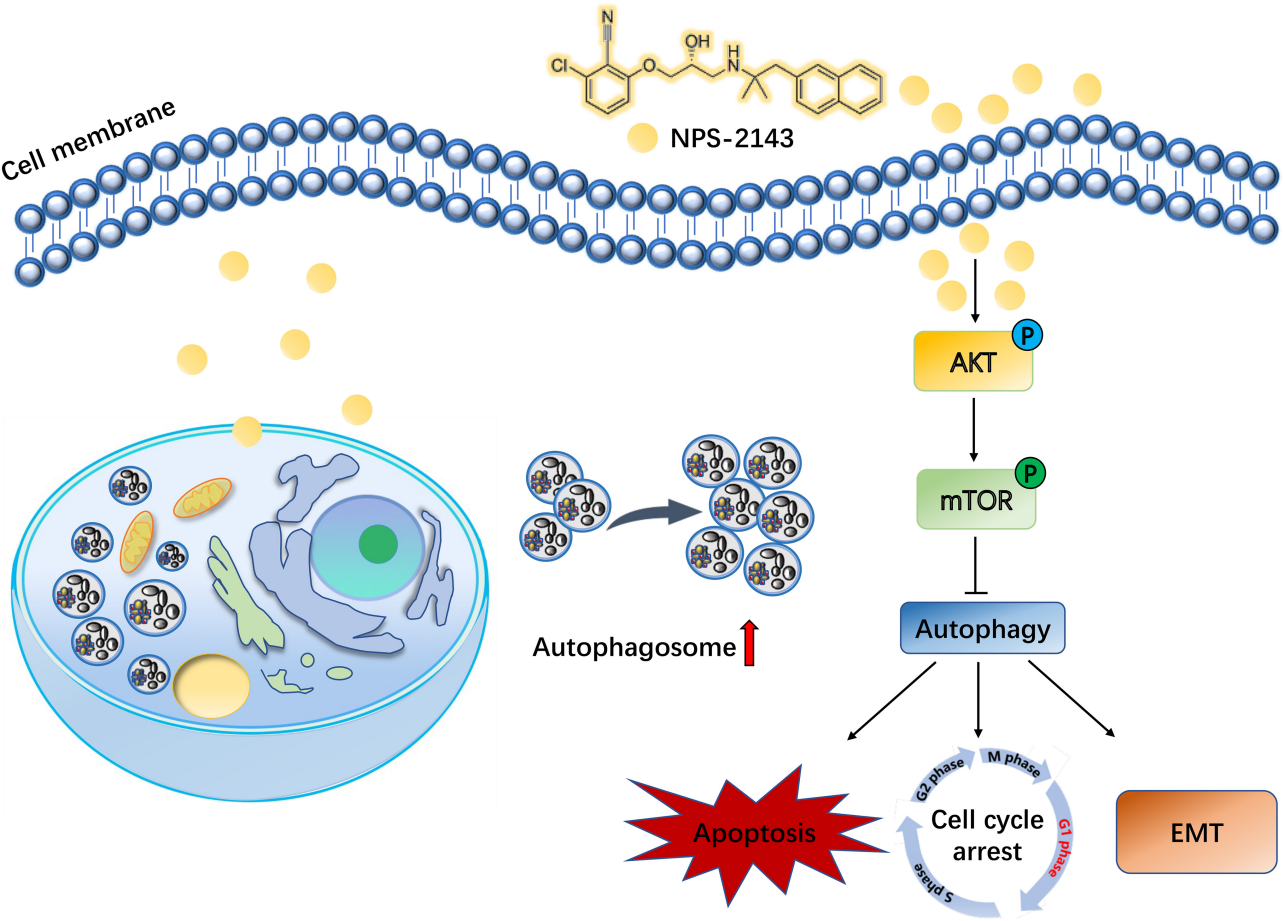
NPS‐2143 suppressed glioma progression by inhibiting autophagy through mediating AKT–mTOR pathway.

In conclusion, we demonstrated that NPS‐2143 could induce apoptosis and cell cycle arrest and inhibit migration of glioma cells by inhibiting autophagy through the AKT–mTOR pathway (see Figure 7).

## AUTHOR CONTRIBUTIONS

**Jia‐Li Nie:** Data curation (lead). **Qi Li:** Software (equal). **Hai‐Tang Yin:** Data curation (equal); software (equal). **Ji‐Hong Yang:** Writing – review and editing (equal). **Ming Li:** Writing – original draft (equal); writing – review and editing (equal). **Qin Li:** Software (equal). **Xing‐Hua Fan:** Software (equal). **Qing‐Qing Zhao:** Software (equal). **Zhi‐Peng Wen:** Conceptualization (lead); writing – original draft (lead).

## FUNDING INFORMATION

This work was supported by National Natural Science Foundation of China (No. 82060667); Guizhou Provincial Science and Technology Projects (No. ZK [2021] General‐563); The Cultivate Project 2020 for National Natural Science Foundation of China, Affiliated Hospital of Guizhou Medical University (No. gyfynsfc [2020]‐34).

## CONFLICT OF INTEREST STATEMENT

The authors declare that they have no conflicts of interest.

## Data Availability

The data sets generated and analysed during the current study are available from the corresponding author on reasonable request.
